# The Hospital of the Future

**Published:** 1916-09-30

**Authors:** 


### The Hospital of the Future.

An address on "The Hospital of the Future," delivered by Dr. C. L. Bonfield, visiting physician at Good Samaritan and Christ Hospitals, Cincinnati, is reported in *Hospital Management*, Louisville. Dr. Bonfield thinks that in hospital construction usability and comfort are of more consequence than architectural elaboration and the use of costly materials. In building the hospital of the future, he says, care will be taken to obtain the desired results at as small an initial cost as possible, and to plan it so that it can be operated at the least possible expense. "Experience has taught that with modern methods of ventilation and with fire-proof construction, there is no reason why a hospital should not be five, six, or even more stories high, if need be, and the necessity of the detached pavilion no longer exists." "If possible the hospital should be so constructed that every room for a patient will some time during the day get sunshine. Sunshine is not only a health-giving agent, but it does much to cheer the patient. The halls and floors should be as near sound-proof as possible, and the doors should be wide enough for a bed to pass through easily." Dr. Bonfield says the kitchen of the future will be in the basement because of the elevator expense of having it on the top floor.

